# Double transition of young migrants in Luxembourg: vulnerable, resilient and empowering integration trajectories in the period of youth

**DOI:** 10.3389/fsoc.2024.1230567

**Published:** 2024-05-10

**Authors:** José Oliveira, Birte Nienaber, Jutta Bissinger, Amalia Gilodi, Catherine Richard, Isabelle Albert

**Affiliations:** ^1^Department DGEO, University of Luxembourg, Luxembourg, Luxembourg; ^2^Department DBCS, University of Luxembourg, Luxembourg, Luxembourg

**Keywords:** young migrants, integration, vulnerability, resilience, empowerment

## Abstract

Migrant integration trajectories have become more complex, open, uncertain, and continuously changing, over time. For young migrants, their integration endeavour intersects with their process of transition to adulthood, a double transition that poses additional challenges. Recent theoretical perspectives such as “liquid integration” aim at focusing on the dynamic, processual, and temporal nature of migrant integration. The present article focuses *on the dynamic interplay of obstacles and enablers that, over time, interact to construct complex, often non-linear, and open-ended integration and coming of age trajectories of young migrants* (aged from 18 to 30 years) coming from outside the European Union (EU) to EU countries. Empirical results from the H2020 MIMY (Empowerment through liquid Integration of Migrant Youth in vulnerable conditions) research project in Luxembourg will be presented. In order to address the goal of the research, qualitative data were gathered by means of *N* = 38 interviews with young migrants with different migratory paths, characteristics and experiences, and specifically included: young migrants from non-EU Portuguese-speaking countries (*N* = 16), refugees living in reception centres (*N* = 15), migrants who since arriving in Luxembourg have become publicly visible (*N* = 7). Content analysis of the interviews allowed a twofold purpose: (1) capturing the unfolding of intersectional integration obstacles that over time play a decisive role in the building of conditions of vulnerability of the double transition under analysis; (2) capturing the multidimensional resources that interactively build up to give rise to resilient and empowering integration and coming of age experiences. The identification of decisive multidimensional obstacles and resources present in the integration endeavour during the process of coming of age allowed us to capture differentiated routes of vulnerability, on the one hand, and resilience/ empowerment on the other. Key ingredients of both *vulnerable* and more *resilient* and *empowering* integration and coming of age trajectories are identified as well as their relational dynamics, enabling to address key challenges for the resilience and empowerment of young migrants in the process of negotiating their transition to adulthood amidst their integration challenges in the Luxembourgish society.

## Introduction

The period of youth is a critical stage in a person’s life that shapes the development of the adult identity, attitudes, goals, and aspirations. It is influenced by the prevailing contextual conditions, attitudes and values of a given society and has a reciprocal relationship with the society’s development. During this time, relationships with peers become more important, while parents remain an essential source of support—especially family-oriented and interdependent cultures tend to maintain strong parent–child relationships and mutual obligations (e.g., Scabini et al., 2006). It is a time when the process of transition to adulthood takes place, encompassing among other major challenges the school-to-work transition and the process of individuation from the family of origin (e.g., Côté, 2014; Oliveira et al., 2014; Arnett, 2015). In recent times, an increased delay in the assumption of adult roles and responsibilities has been observed. This phenomenon has led to the proposal of a new developmental phase situated between adolescence and young adulthood, which was named *emerging adulthood* (Arnett, 2000, 2015). This new phase would possess the following characteristics: Extensive processes of identity exploration, trying to answer the question “who am I?” and experiencing various options and responses, especially in the domains of love and work; considerable amount of instability, especially in the domains of work, love relationships, and places of residence; a preponderance of a strong focus on oneself, since obligations towards others are markedly scarce; strong feelings of being in an intermediate phase of life, a sense of being “in-between” adolescence and adulthood (giving young people a feeling of being in transition, not identifying themselves as adolescents, nor yet as adults); the perception of living a phase of possibilities characterised by great optimism about future life trajectories (Arnett, 2000, 2015).

Notwithstanding, emerging adulthood has, from the very beginning, remained a contested perspective on present day challenges and complexities inherent to the process of transition to adulthood (e.g., Côté, 2006, 2014; Côté and Bynner, 2008; Hendry and Kloep, 2012; Côté and Levine, 2014). In contrast to the celebratory tone of the emergence of a new developmental phase full of optimism and possibilities for the future, typical of the emerging adulthood perspective, one of its most vocal critics, Côté (2014), presents a more sombre perspective on the changes occurring in the process of transition to adulthood. Designating the emerging adulthood theory as a dangerous myth (born of logical and empirical fallacies) that naturalises the difficulties currently experienced by young people in their transition trajectories to adulthood, Côté proposes that we are facing an extension of the psychosocial moratorium for identity development that has been widened to a growing segment of the young population (Côté, 2006). A fact arising from structural obstacles such as those linked to difficulties in integrating into the world of work (construction of occupational identity) and in obtaining financial independence.

Whether one is witnessing the emergence of a new developmental stage in the life course or whether the delay in the assumption of adult roles and responsibilities is a result of contextual factors (e.g., difficulties in entering the labour market, prolongation of school trajectories, the financial impossibility of accessing autonomous housing arrangements, ensuing dependency from the family of origin and delayed co-habitation; Côté, 2006, 2016), it is evident that coming of age trajectories have become more complex, non-linear, and protracted in time. This multifaceted journey is marked by significant changes and challenges, in present-day Western societies, where young individuals encounter a unique set of obstacles as they navigate this pivotal phase of life, in light of the societal, economic, and cultural factors that shape the experiences of young emerging adults. One of the foremost challenges facing young emerging adults in Western societies is *economic uncertainty*. Factors such as rising housing costs, stagnant wages, and increasing student debt contribute to financial instability (Arnett, 2015). The traditional markers of adulthood, such as homeownership and stable employment, are increasingly out of reach for many young people (Settersten and Ray, 2010). Economic pressures can delay key life decisions, such as marriage and parenthood, as individuals prioritise financial stability over other milestones. In terms of *education and employment*, the transition from education to employment presents a significant hurdle. Despite obtaining higher levels of education than previous generations, many young people struggle to secure meaningful employment (Schoon and Bynner, 2017). The prevalence of precarious work, characterised by temporary contracts and low wages, exacerbates this challenge (Standing, 2014). Furthermore, the mismatch between the skills acquired through education and those demanded by the labour market contributes to unemployment and underemployment among young adults (Hussmanns, 2010). Navigating the complexities of the job market requires resilience, adaptability, and often additional training or re-skilling. Furthermore, the previously mentioned challenges occur in a social and cultural context marked by profound *social and cultural transformations* that impact the transition to adulthood. Changing family structures, increased diversity, and evolving gender roles contribute to a shifting landscape of social expectations (Furstenberg, 2010). Young adults grapple with the pressure to balance individual autonomy with familial obligations and societal norms (Arnett, 2004). Moreover, the pervasive influence of digital technology and social media shapes social interactions, identity formation, and perceptions of success (Twenge, 2017). Navigating these dynamics requires critical thinking skills, self-awareness, and a strong sense of identity.

Such economic, social and cultural challenges may have a huge impact on the mental health and well-being of emerging adults. The transition to adulthood has become a period of heightened vulnerability for mental health issues in Western societies. Young adults face stressors such as academic pressure, financial worries, and uncertainty about the future (Collins and Klerman, 2011). Moreover, the stigma surrounding mental health can deter individuals from seeking help, exacerbating the problem (Hunt and Eisenberg, 2010). Addressing mental health challenges requires destigmatization, access to affordable and culturally competent care, and proactive support systems within families, schools, and communities. Overall, the transition to adulthood in present-day Western societies is fraught with challenges that encompass economic, educational, social, and mental health dimensions. Economic uncertainty, education-to-employment transitions, social and cultural shifts, and mental health concerns shape the experiences of young adults as they navigate this pivotal phase of life. Recognising these challenges is essential for policymakers, educators, and community leaders to develop effective strategies and support systems that empower young people to thrive in an ever-changing world.

Young people who have left their countries of origin and are now in the process transition to adulthood (aged 18–29) face the added challenge of going through the micro-transitions to adulthood (e.g., finishing their studies, entering the labour market, eventually forming their own family with the assumption of conjugal and parental roles) in the context of migration. This ‘double transition’ (King and Koller, 2015) also differentiates them from other migrants. Young migrants are motivated to leave their home country for a variety of reasons such as to secure a better future (for themselves and their families), to escape war or harm, to pursue their educational aspirations, for family reunification (e.g., Di Cristo and Akwei, 2023). During their migration journey, some migrants may already start experiencing an array of multiple difficulties: especially young migrants may be at a higher risk of abuse, trafficking and other forms of exploitation (UNICEF, 2017). Upon arrival in the host country, integration challenges ensue for many migrants, related to the process of adaptation to a new cultural environment, to the absence of social ties, to difficulties in finding appropriate housing arrangements, and many other challenges for which suitable coping strategies have to be found (e.g., Bauböck and Tripkovic, 2017).

One of the foremost challenges migrants face is *cultural adjustment*. Moving to a new country entails adapting to unfamiliar customs, traditions, and societal norms. This adjustment can be particularly daunting during the formative years of adulthood when individuals are still shaping their identities. Unlike their peers in their home countries, migrants must navigate the complexities of acculturation, grappling with issues of belongingness and identity fusion (Berry, 2005). The clash between their native culture and the host country’s culture often presents dilemmas, leading to a sense of cultural dissonance. Language proficiency may constitute another significant challenge and determinant of successful integration into a new society. For migrants, mastering the language of their host country poses a formidable challenge. Limited language skills hinder communication, impede educational attainment, and restrict employment opportunities (Chiswick and Miller, 2004). In contrast, individuals transitioning to adulthood in their own countries are likely fluent in the predominant language, facilitating seamless communication and social interaction.

Added challenges are brought forward by expected difficulties in establishing social networks and support systems, which are of paramount importance in the period of transition to adulthood. Migrants often encounter difficulties in building robust social connections due to factors such as cultural differences, discrimination, and geographical isolation (Luthar and Ansary, 2005). Unlike individuals in their home countries who may rely on familial and community ties for support, migrants may lack such networks, exacerbating feelings of loneliness and alienation.

Legal and administrative challenges may also pave their path to adulthood. Navigating the legal and administrative frameworks of a new country can be overwhelming for migrants transitioning to adulthood. Issues such as visa regulations, residency permits, and unfamiliar bureaucratic processes add layers of complexity to their journey. Moreover, undocumented migrants face heightened vulnerability, with limited access to essential services such as healthcare and education (Bloemraad et al., 2008). In contrast, individuals in their own countries are typically familiar with the legal system, making the transition to adulthood relatively smoother.

As a result of the above-mentioned challenges or as a challenge on its own, economic struggles often characterise the path to adulthood of young migrants. Many migrants face precarious employment conditions, low wages, and limited career prospects, hindering their financial independence and upward mobility (Dustmann and Weiss, 2007). Economic challenges intersect with other facets of migration, exacerbating social exclusion and perpetuating cycles of poverty.

Such challenges occur at a time in their lives when they are making their transition to adulthood (Titzmann and Lee, 2022). Hence, their already challenging trajectories of becoming independent from their families and of finding a satisfying transition into the world of work may be further burdened by the added complexities of having to fulfil these developmental tasks as migrants subject to integration strains that may be extremely demanding.

Young migrants are a dynamic group that is transitioning from education to work, and their experiences during this time can have a profound impact on their future. From a socio-economic perspective, the period of youth is crucial as any disruption in the transition from school to work can lead to the social and economic exclusion of young people, which has long-term consequences both for the individuals and for society as a whole (e.g., Schoon and Bynner, 2019). Young individuals, particularly young migrants, may be especially prone to fall into vulnerable situations, stemming, among other sources, from difficulties in the process of acculturation, eventual lack of sufficient family support, difficulties associated to the complex school-to-work transition that may heighten the possibilities of landing in unemployment or NEET (not in Education, Employment, or Training) situations or only being able to attain precarious job positions (e.g., Kosidou et al., 2012). With respect to job integration, employment levels for individuals between the ages of 15–29 have systematically been relatively low in many European countries, and there is generally an increased risk of poverty for young people (Eurostat, 2023).

Based on these concerns, the H2020 project MIMY (Empowerment through liquid integration of migrant youth in vulnerable conditions), set out to gain a deeper understanding of the needs of young non-EU migrants living in vulnerable conditions between the ages of 18 to 29, as well as to capture ingredients and triggers of resilient and empowering integration trajectories during the period of transition to adulthood, by analysing 18 local case studies in 9 European countries,. This article presents findings from the Luxembourgish case studies. Luxembourg is a small European state with a population of 645,397 people, as of January 2022, of which almost half (47.1%) do not hold Luxembourgish citizenship (STATEC, 2022). This composition makes Luxembourg an interesting case study to analyse more in depth the complex integration experiences of migrants. In Luxembourg, the majority of the foreign population is from other EU countries. The biggest communities are the Portuguese (14.8% of the country’s residents), the French (7.6%), and the Italian (3.7%).

Indeed, with the development of the coal and steel industry migrants were recruited from Italy, Germany, and later Portugal to satisfy the labour demand. In the 1980s and 1990s, Luxembourg changed from an industrial economy to today’s global financial economy which led to a more diversified migrant population, as the country is especially in need of high-skilled migrants recruited from all over the world (Kolnberger and Koff, 2021). Additionally, the number of asylum applications increased in recent years, especially from Syria, Eritrea, and Afghanistan. Thus, the number of third-country nationals (TCNs) living in Luxembourg has been increasing within the last two decades (STATEC, 2022) and in 2021, they represented 8.6% of the total population (EMN, 2021). In terms of their living conditions, integration indicators provided by Eurostat (2023) show that TCNs in Luxembourg are at higher risk of poverty and social exclusion (36.5% compared to 15.4% for nationals), early school leaving (32.4% compared to 7.0% for nationals), and unemployment (12.3% compared to 4.2% for nationals). Furthermore, Luxembourg is a multilingual country with three official languages (Luxembourgish, French, and German) and English becoming an important working language. This aspect of the Luxembourgish context is relevant to consider since multilingualism is a factor that may add substantial difficulty and complexity to the integration of newly arrived migrants in the country in several spheres: socialisation, school or work integration, contact with public services, cultural adaptation.

### An eco-developmental and multi-dimensional approach to integration: “liquid integration”

The MIMY project departed from the traditional notion of the concept of integration. Even though extensive theoretical and empirical research has been done in various fields over decades on integration, the concept remains contested in normative and theoretical discourses (Ager and Strang, 2008; Wieviorka, 2014; Rytter, 2018; Schinkel, 2018; Biaback Anong et al., 2023). The focus is frequently set on migrants adapting to the cultural and economic norms of the host country and the perspectives of migrants are often missing (Erdal, 2013; Cederberg, 2014; Valsiner, 2022). The MIMY project aimed to promote a more nuanced and comprehensive understanding of integration by drawing on the concept of “liquid integration” (Skrobanek and Jobst, 2019) focusing especially on the perspective of young migrants, and emphasising the complex and evolving nature of integration, which involves multiple interdependencies at various levels, including macro, meso, and micro. Furthermore, it takes into account time and space, viewing integration as a lifelong exchange and adjustment between individual and context. Liquid integration derives from Bauman’s concepts of “liquid modernity” and “liquid society” (Bauman, 2001, 2007) and Engberson’s concept of “liquid migration” (Engberson, 2012) combined with the notion of longitudinal development (Skrobanek and Jobst, 2019). Within the liquid integration perspective, migrant integration is viewed as having an open shape (with no precise beginning and end), constituting a permanent multidimensional process of adjustment to new contexts and the ongoing change of the latter. Liquid integration, thus, denotes a life-long dynamic interdependency and interplay of agency and structure (Skrobanek and Jobst, 2019).

### Liquid integration and the processual construction of vulnerability, resilience and empowerment

The analysis rests mainly on the concept of liquid integration as the pivotal theoretical framework. Yet, in order to capture the direction of liquid integration and coming of age paths, we draw on a secondary set of concepts that will allow the characterisation of different integration trajectories. Such concepts consist of *vulnerability*, *resilience* and *empowerment*. These concepts and the way in which they will be understood and approached in this article will now be presented.

Young migrants settling in a new country and navigating their *liquid integration* journeys are inevitably confronted with numerous challenges and difficulties that the literature on integration has been identifying. Identified stressors are manifold: Uncertainties associated with asylum procedures and legal status, experienced problems in accessing services, difficulties in job integration, economic difficulties and insufficient resources to support oneself and one’s family, challenges associated with finding adequate housing, social isolation, differing levels of welcoming practises and attitudes from the receiving community that may entail discrimination, adapting to new cultural values, lifestyles, languages and policies, lack of social recognition, minority status, coping with disadvantage, stigma and emerging psychological disorders (e.g., Ward, 2001; Fazel et al., 2005; Sam and Berry, 2010; Bronstein and Montgomery, 2011; Salazar and Jayaram, 2016; Papadopoulos and Gionakis, 2018; Schwartz et al., 2018; Ciaramella et al., 2022; Gilodi et al., 2023).

Accordingly, and in order to tackle widespread integration difficulties faced by migrants, the concept of *vulnerability* has recently started to be used as a way to address the manifold difficulties inherent to the processes and trajectories of integration in a given host society (e.g., Bates-Eamer, 2019; Hoefinger et al., 2019; Gilodi et al., 2023). Yet, the task of defining a clear, comprehensive, and useful concept of vulnerability is not without its challenges (Gilodi et al., 2022). In fact, vulnerability has often been presented as a self-explanatory condition in previous literature, which has hidden its complexity, and limited its analytical power (Gilodi et al., 2022). Additionally, varying conceptualizations and uses of vulnerability in social and migration policies, which may include approaches to migrants’ integration, may have a host of deleterious effects for the migrants who are subjected to them: they can be discriminating, stigmatising, patronising, disempowering, may foster exclusion, social control and even oppression (Gilodi et al., 2022).

In an effort to clarify the concept of vulnerability in the context of migration and render it analytically useful and effective, Gilodi et al. (2022) offered a new and comprehensive conceptual model of vulnerability presenting it as multi-layered, dynamic, and embedded in specific (and changing) socioeconomic, political, historical and cultural contexts. In such a conceptualization, vulnerability is not reducible to some fixed, measurable condition or set of conditions that are immutable throughout time and space, nor merely susceptible to being described as innate, situational or structural (Gilodi et al., 2022). Imbued with an ecological, developmental and systemic perspective, such an approach seems to enable the capturing of the dynamic nature of vulnerability, by pointing to its embeddedness in existing socio-political hierarchies with ongoing power dynamics. The latter are constantly being played out, interpreted and negotiated within local systems and interpersonal relationships, at different points of the individuals’ life course (Gilodi et al., 2022). As a consequence, the experience of vulnerability is to be individually assessed, contextualised in a specific space and time, at a specific point in the life course, and is viewed as a result of a continuous dynamic interplay of “structural, situational, social, biographical, and psychological characteristics” (Gilodi et al., 2022; Arendas et al., 2023).

From a political perspective, and in an attempt to overcome the defy of producing a clear and operational definition of vulnerability, the United Nations (UN), offered the broad definition of “migrants in vulnerable situations” as “persons who are unable effectively to enjoy their human rights, are at increased risk of violations and abuse, and who, accordingly, are entitled to call on a duty bearer’s heightened duty of care” (United Nations, 2018, p. 5). In this sense, the UN points to the fact that vulnerability must not be understood as something inherent to the condition of migrant and associated to an intrinsic lack of resilience and agency. Contrariwise, the UN looks at vulnerability as both *situational* and *personal* (United Nations, 2018): a condition that must be assessed individually and a “result of multiple and intersecting forms of discrimination, inequality and structural and societal dynamics that lead to diminished and unequal levels of power and enjoyment of rights” (United Nations, 2018, p. 6).

In light of such recent theoretical and institutional developments regarding the conceptualization of vulnerability, in this study a comprehensive approach was adopted which enabled to encompass various dimensions such as negative life events, adverse childhood experiences, illnesses, injuries, and disabilities (Barocas et al., 1985), as well as social, cultural, and economic exclusion (Bhalla and Lapeyre, 1997; Ligon and Schechter, 2003). Moreover, when examining the situation of vulnerable migrant youth, we refrained from imposing a preconceived definition of vulnerability. Instead, it was assumed from the onset that migrants themselves are best equipped to identify the areas in which they feel vulnerable, whether it be in regard to health, the labour market, education, political participation, or other areas. It was further acknowledged that migrants may have a distinct perspective and may not view themselves as vulnerable in areas where they appear to be so according to statistical measures or outward social criteria, and vice versa.

When talking about migrants’ vulnerability, it is almost inevitable to bring to the fore the concept of *resilience* as a kind of complementary or counterpoint force to experienced conditions of vulnerability (e.g., Aroian and Norris, 2000; Udah et al., 2019). Along with the systemic, ecological and developmental approach to vulnerability, a similar approach to resilience seems to be in order when addressing migrants’ integration challenges. These integration challenges may stem from multiple structural, contextual and individual factors, at play at a specific point of the migrant’s life course, therefore they ask for the interplay of multidimensional resilience resources and strategies, from more broadly socio-structural to more individual in nature.

Hence, in order to overcome the multiplicity of vulnerable conditions young migrants may face, a host of multidimensional resilience factors are called to the fore to respond to the manifold challenges they face in their integration trajectories. Resilient integration trajectories thus demand a host of resources and actions that span from macro contextual dynamics to micro individual responses to everyday challenges. Moreover, the building of young migrants’ resilience is assumed to have both a systemic and a developmental nature. By systemic, we point to the fact that there is a continuous interplay between structural conditions and agentic resources (e.g., Masten, 2021) in the construction of resilient integration paths. On its turn, the developmental character of resilience addresses the unfolding construction of resilience resources and strategies (Masten, 2014, 2021) over the course of time within the context of integration paths, conceived as open-ended and spanning along the life course. Such a complex and systemic conception of resilience is becoming increasingly prevalent in the literature (e.g., Ungar, 2018; Zabaniotou, 2020; Hayes, 2022).

Using a developmental systems perspective, Masten (2021, p. 116) has defined resilience as “the capacity of a dynamic system to adapt successfully to challenges that threaten the function, survival, or development of the system.” Far from a merely individual capacity, the systemic approach to resilience encompasses interdependent factors and processes, scalable across system levels, from the macro (socio-structural) to the micro (individual) level. Moreover, its developmental nature points to the fact that the resilience of any living system, such as a person, is closely associated to particular and individual processes of change and development (and eventual decline) that occur throughout the life course and that have an impact on the systems’ adaptive capacity (Masten, 2014,; Masten and Cicchetti, 2016). Accordingly, when faced with life challenges, the need and eventual capacity of the living system to establish a new equilibrium, a new balance required to resume the system’s developmental path, becomes salient.

Although resilience plays an enormously important role in the development of successful integration trajectories, our study went further and aimed at exploring the construction of *empowering* integration trajectories. Issues of power (or lack thereof), as we have seen above in the UN’s definition of vulnerability, are at the heart of vulnerability. According to Verbena et al. (2022), resilience and empowerment present both similarities and differences. In terms of similarities, both concepts point to strength-based approaches aimed at promoting positive outcomes. Yet, resilience stresses processes of positive adaptation to contexts that present significant adversities or risks (Luthar and Cicchetti, 2000), while empowerment addresses any shifts in power that have the effect of producing gains in terms of influence within social relationships and the broader social context (Cattaneo and Chapman, 2010). In other terms, the focus of change in the two approaches is different: in the case of resilience, the focus of change is internal, whereas in the case of empowerment, such a focus is external. Thus, resilience refers to intrapersonal actions leading to internal changes such as the capacity for adapting, resisting or overcoming risks and challenges. Empowerment, being externally oriented, is directed to producing changes in inter-relational and community power dynamics (accompanying the more internal or more psychological shifts; Kenny, 2022; Kenny and Young, 2022; Verbena et al., 2022). Another differentiating aspect between resilience and empowerment is associated to context or circumstances. Whereas resilience always occurs in a context of risk or threat, empowerment may also occur in non-risk circumstances (Brodsky and Cattaneo, 2013). As such, the distance between the baseline condition and aspired goals in risky situations is usually greater than in a context where risk and threat are absent (Brodsky et al., 2022; Verbena et al., 2022).

Yet, given the similarities of both approaches (both aiming at strengthening agentic capacities) and their substantial overlapping, Brodsky and Cattaneo (2013) have proposed a Transconceptual Model of Empowerment and Resilience (TMER) with the objective of identifying shared resources and processes. TMER specifically focuses on “internal and external resources (or lack thereof) in immigrants’ everyday lives; individual-community relationships as they unfold at the social, cultural, and material levels; goals and actions chosen by immigrants to respond to these conditions; and the divergence (or convergence) of those goals and actions in resilience and empowerment processes” (Brodsky and Cattaneo, 2013; Brodsky et al., 2022). This model therefore may be appropriate to comprehensively tackle the development of resilient and empowering migrant integration trajectories, while also differentiating resilience and empowerment (Brodsky et al., 2022). Within the TMER model, resilience and empowerment are conceptualised as iterative and interactive, occurring at different ecological levels and having fluid boundaries. As such, it acknowledges that people can shift from one to the other, depending on available contextual resources (Verbena et al., 2022). TMER has recently been applied in the context of migration studies as well as contexts of discrimination to account for fluid processes of resilience and empowerment (Brodsky and Cattaneo, 2013; Godsay and Brodsky, 2018; Buckingham and Brodsky, 2021; Brodsky et al., 2022; Verbena et al., 2022). In the present study both resilience and empowerment will be examined in accordance with TMER in order to account for the intersectionality and interactivity between resilience and empowerment strategies and processes.

### Rationale and aims of the study

The present research aimed at analysing the integration experiences of young migrants in Luxembourg, taking into account their diverse biographical backgrounds and the multiple challenges they face. For that purpose, two groups of migrants in vulnerable conditions (one composed of young migrants coming from non-EU Portuguese-speaking countries in precarious job conditions, and another composed of refugees living in reception centres) and a group of young migrants with public visibility were included in the study. The two first groups are constituted of young migrants in vulnerable conditions: in the first group, stemming from precarious job situations; and in the second group, stemming from their condition of holding refugee status but living in reception centres for asylum seekers. The third group includes young migrants with public visibility, thus appearing to have achieved a certain degree of empowerment in their integration process that granted them public notoriety in diverse fields. The aim was to examine processes of vulnerability affecting all groups of migrants (but focusing especially on the first two signalled groups in vulnerable situations) and on processes of resilience and empowerment, expected to be especially visible in the last group under study (although not solely, as these processes may be also present in the first two groups). The study addressed forms of vulnerability stemming from multidimensional obstacles to the integration process, as well as available resources and strategies that may have a positive impact in building resilient and empowering responses to experienced difficulties and challenges, and that foster positive integration trajectories. Due to expected multiple forms of vulnerability associated with different biographical backgrounds, also variable forms of dealing with experienced challenges were expected. The diverse tools and strategies that can help to foster young migrants’ resilience and empower them to become active in the host society were thus examined. The overarching aim was to explore the possible different ingredients present in vulnerable, resilient and empowered liquid integration trajectories during the period of coming of age and observe their presence or absence in the narratives of the three groups under study (thus accounting for group differences and commonalities). By contrast with the existing literature, which tends to focus on specific aspects of the integration of migrants, the present study aims to jointly address the double transition they face (integrating into a new society while transitioning to adulthood), drawing on the analytical potentialities of the liquid integration perspective and focusing on the processual construction of, on the one hand, vulnerable transition trajectories and, on the other hand, on resilient/ empowering transition trajectories.

## Materials and methods

In order to capture, on the one hand, vulnerability conditions and processes, and, on the other, the processual nature of resilience and empowerment, semi-structured individual interviews, lasting between 30 and 130 min, with young migrants were conducted within the framework of the MIMY project, in Luxembourg. Interviewees were recruited through various means such as with the assistance of institutions working in the field of migrant integration and youth associations. The interviews addressed the following themes: exploration of significant events that represented a change in the interviewees’ life; exploration of vulnerability and resilience at the individual level (e.g., personal weaknesses and strengths); exploration of vulnerability and resilience at the family level (e.g., quality of family relationship and perceived support); exploring of socialisation and educational paths; exploring the relationship with the labour market (e.g., past and present job experience, personal skills and main challenges to find a job or change a job, future planning/projects to improve own job condition); exploration of the financial and housing conditions; exploration of vulnerability and resilience considering the social and intergroup relationships (contact, quality of relationships, trust and closeness with the local population); exploration of vulnerability as a label; exploration of future plans and perspectives. Empowerment was explored through the analysis of the levels of sociopolitical and community participation of interviewees.

During the period of November 2021 and February 2022, the three different groups of young non-EU migrants were interviewed in person as well as online: economic migrants from Portuguese-speaking countries in precarious job situations, refugees still living in reception centres for asylum seekers, and young migrants with public visibility. The first two groups were selected as, based on certain characteristics, they appeared to be in particularly difficult or precarious conditions in the specific context of Luxembourg. The third group was selected as potentially resilient, empowered and structurally integrated, as they were often regarded as ‘success’ storeys given their role as publicly visible migrants. Starting from these external selection criteria, the interviews aimed at exploring the young migrants’ personal experiences and expectations in different areas of integration (e.g., family, education, work, housing, social contacts) in order to capture the construction of vulnerabilities as well as resilient and empowering processes and how they related to different trajectories. Interviews were conducted with young migrants’ living both in the north and in the south of the country in order to capture their diverse experiences, irrespective of geographic location, thus overcoming potential regional specificities.

The first group, non-EU Portuguese-speaking economic migrants in precarious job situations were interviewed in the South (10 interviews, 4 male and 6 female, aged between 21 and 30 years, 5 coming from Cape Verde and 5 from Brazil) and North (6 interviews, 2 male and 4 female, aged between 19 and 28 years, 4 coming from Cape Verde and 2 from Brazil) of Luxembourg. The length of their stay in Luxembourg ranged from 1 to 11 years. This group was chosen given the number of non-EU migrants from Portuguese-speaking countries in Luxembourg increased in the last decades due to the connection with Portugal and as Portuguese migrants being the biggest group. More specifically, Cape Verdean migrants started arriving in the country in the 1970s and have shown steady inflows since then, being presently the third African country with most inflows (STATEC, 2022). In the last years, the inflow of migrants from Brazil has been increasing considerably, having made to the top 10 countries with most inflows (STATEC, 2022).

The second group, refugees, still living in reception centres, were interviewed in the South (9 interviews, 6 male and 3 female, aged between 19 and 28 years, 2 coming from Afghanistan, 3 from Eritrea, 3 from Syria and 1 from Iran) and North (6 interviews, all males, aged between 20 and 30 years, 3 coming from Afghanistan, 2 from Iraq and 1 from Eritrea) of Luxembourg. The length of the refugees stay in Luxembourg ranged from 7 months to 6 years and 1 month. This group was selected as even though they have obtained refugee status, they are not able to move out of reception centres due to the difficult housing situation in Luxembourg. Almost half (44.4% as of the end of 2021) of the people living in reception centres in Luxembourg already hold a refugee status but are not able to find other housing arrangements (Gilodi et al., 2023).

For the third group, 7 young non-EU migrants with public visibility living in Luxembourg, irrespective of their place of residence, were interviewed (3 male and 4 female, aged between 18 and 32 years, 3 from Iran, 1 from Syria, 1 from Somalia, 1 from Canada, 1 from India). The migrants were selected based on their public visibility in Luxembourg in different contexts (politics, activism, business, research, and sports). The selection criteria, public visibility, was chosen to examine in more detail their personal integration experiences and to verify if their public visibility represents a more positive integration experience, when compared with the two previous groups of young migrants in vulnerable conditions. The interviews with this group focused on the factors of resilience and empowerment that could favour the positive experiences of young migrants, despite the difficulties encountered. The participants represent a diverse group coming to Luxembourg from different non-EU countries, having arrived alone or with their families, and for diverse reasons (e.g., to ask for asylum, to study, to work). The length of their stay in Luxembourg ranged from 4 to 10 years. Depending on the context of activity (politics, activism, business, research, sport), the young migrants benefitted from different social contacts and support systems.

**Table 1:** Participants belonging to the three groups under study, according to age and gender.

Group	Age range	N - Male	N - Female	N - Total
Portuguese-speaking non-EU migrants	19–30	6	10	16
Refugees	19–30	12	3	15
Migrants with public visibility	18–32	3	4	7
Total	18–32	21	17	38

The whole sample comprises 38 interviews with young non-EU migrants (Table 1). Before conducting the interviews, ethical concerns regarding vulnerable populations were discussed in detail with the internal ethical board of MIMY, and the ethical approval was obtained by the Ethical Committee of the University of Luxembourg. Different information sheets and consent forms were created to adapt to the specific needs of each group. The interviews were conducted in several languages, namely French, English, Portuguese and German, in order to accommodate the interviewees’ language preferences.

A collaborative thematic analysis of the data (e.g., Braun and Clarke, 2006) was carried out by the researchers in order to identify, analyse and report themes, their patterns and interrelations. The interviews were analysed using the MAXQDA^©^ software. Codes were created both deductively (from expected themes already present in the existing literature) and inductively, thus remaining open to the own experiences and perceptions of the interviewees. At first, open codes were used, and then main codes were developed. The interviews were first analysed per group and then compared. Taking into account the similarities found between groups, results were grouped according to main themes found (in terms of vulnerability and resilience/ empowerment). Group-specific themes were kept with reference to the group where they were mentioned.

## Results

Results will be presented in accordance with the research’s goals: (1) To scrutinise the processual and cumulative effect of barriers to integration in the construction of vulnerabilities; (2) To examine the processual unfolding of factors and triggers of resilient and empowering integration trajectories. Group specificities (according to the experiences of the three groups of migrants under study—young migrants from non-EU Portuguese-speaking countries, young refugees, and young migrants with public visibility) will be mentioned whenever observed. Names of participants associated to each quote were substituted by pseudonyms.

### The cumulative effect of barriers to integration in the construction of vulnerabilities

Results pertaining to the construction of vulnerabilities allowed us to observe that they usually stem from various structural and situational conditions pertaining to several dimensions: legal barriers, housing, labour market integration and the high cost of living of the country, lack of family support, social barriers and cultural differences, multilingualism. Results will be presented along the latter dimensions highlighting their intersectionality and interdependence in the construction of paths of vulnerability (see Table 2 for a summary of reported challenges and barriers young migrants face, according to the groups under study).

**Table 2:** Challenges mentioned by young migrants according to the groups under study.

Challenges and barriers	Challenges and barriers effects
Legal barriers	***Migrants from Portuguese-speaking countries*** Legal barriers may lead to a failure to receive a residence permit. Being irregular affects all spheres of life: it does not allow young migrants to find a legal job, or have access to health care, and thus is ultimately affecting the young migrant’s quality of life. ***Refugees*** In the case of refugees, they may be left in a limbo, living with limited rights (such as the right to cross borders and limited access to the labour market) and with uncertainty about their future. In some cases, this protracted phase of uncertainty can cause feelings of being rejected which may have a negative Effect on future integration trajectories.
Housing	***Migrants from Portuguese-speaking countries*** Oftentimes migrants have only access to small apartments that, when shared, bring the additional difficulty of lack of privacy. ***Refugees*** As for the young people holding refugee status, by study design, they all shared a precarious housing situation. They had been recognised as beneficiaries of international protection (BIP) but were still housed in temporary reception centres, officially designated to accommodate applicants for international protection (AIP), due to the difficult housing market in Luxembourg. Common issues relating to the general overcrowding of the facilities were described by the great majority of the interviewees, such as the lack of privacy, issues of noise and cleanliness.
Labour market integration and the high cost of living	***Migrants from Portuguese-speaking countries*** Difficulties in finding jobs in the industrial sector. Contrary to some migrants’ expectations, finding work in Luxembourg may not be an easy task, which can have a huge impact on their lives: life may be reduced to a process of mere survival (earning just enough money to pay for monthly expenses). ***Refugees*** Independently of whether they were employed or they were studying, the financial situation of the refugees interviewed was overall quite precarious.
Social barriers: discrimination and racism	***Migrants from Portuguese-speaking countries*** Perceived discrimination was reported in everyday life, within the educational system, public services. Discrimination can be based on language or as a manifestation of racism. ***Refugees*** Contrariwise, discrimination and prejudice did not emerge as prominent issues among the refugee group as a whole, especially compared to what they experienced in other countries of residence. Yet, some participants did report feeling negatively judged, discriminated or stigmatised by the local population as refugees and described the overreaching and cumulative negative effect this may have on integration trajectories
Multilingualism	***Migrants from Portuguese-speaking countries, refugees and young migrants with public visibility*** Participants of all 3 groups mentioned the multilingual situation as a barrier to integration. The country has three official languages: Luxembourgish, French and German. Each of these languages may play a significant role in the integration process depending on the region in which the migrant lives, the jobs he/she aspires to, the socialisation demands, access to public services. Language skills are perceived as central to the process of integration in Luxembourg, either for socialization purposes, for work integration or for escaping discrimination and, ultimately, social exclusion.

### Legal barriers

For young migrants in vulnerable conditions coming from non-EU Portuguese-speaking countries, barriers to integration may start with the failure to attain legal or bureaucratic requirements such as a residence permit and therefore being irregular at first. Some migrants from Brazil and Cape Verde have already obtained Portuguese citizenship, and therefore arrive to Luxembourg as EU citizens, instead others arrive undocumented and struggle to receive a residence permit. Being irregular affects all other spheres, and does not allow them to find a legal job, or access to health care, and thus is ultimately affecting the young migrant’s quality of life. As an example, Frederico (male, aged 26, 3 years in Luxembourg), married to a woman from Cape Verde who holds a residence permit and with whom he has a new born child, states that *“Now I am personally experiencing what migrating without documents feels like… you arrive here without documents and you have everything against you, nothing in your favour […].”* He adds that *“What affected me the most and left me depressed was staying imprisoned in the house… I had no daily routine […] you are here but you are not living […] the isolation is huge […] you feel you are in a kind of a prison where you cannot do anything….”* In terms of work, all he could find was irregular work, what he termed “negro work,” a kind of work that is somewhat hidden from local authorities: *“Not having papers what I have to do is ‘negro work’… ‘negro work’ you have to do it after [the regular working hours of] the boss.”* The consequences of such a condition, in terms of mental health, can manifest themselves in terms of anxiety and depressive symptoms: *“I feel that I am nervous all the time, I have no patience for anything… and I was never like this… I’ve always looked at things in the face and now I am feeling low… it’s a situation in which you sometimes even feel like doing some bad things because you feel humiliated.”*

As for the refugees interviewed, one of the main legal barriers encountered was the length of the asylum application process. During this time, these young people are left in a limbo, living with limited rights (such as the right to cross borders and limited access to the labour market) and with uncertainty about their future. In some cases, this protracted phase of uncertainty can cause feelings of being rejected which may affect future integration trajectories, even after asylum was granted, as Aref (male, aged 19, 3 years in Luxembourg) explained: *“… I do not know why, but for us it took a long time. And still some people, they have not got the answers. […] I have a friend when he was coming, he took a profile picture of him with the flag of Luxembourg. He was so honoured with that, [that] he came to Luxembourg and stuff like that. Still, they have not given him the answer. And he detests this country. Hates this country. Just wants to get his status and leave. Just run away. […] because he was disappointed, because he was so eager to do so many things. […] [Now] he just wants to leave.”*

### Housing

Finding affordable housing is a general problem in Luxembourg and was among the biggest hurdles for the great majority of the young migrants interviewed. Among the ones coming from non-EU Portuguese-speaking countries, Tomé (male, aged 20, 10 years in Luxembourg) states that *“housing can be very problematic because, besides its cost, it is difficult to find a house.”* Due to housing costs, oftentimes migrants have only access to small apartments that, when shared, bring the additional difficulty of lack of privacy. As Tomé, living with his family in a small apartment, points out, *“it is quite difficult to have some privacy… I registered in libraries in the city in order to go there when I needed to be alone.”* António, (male, aged 21, 2 years in Luxembourg) goes on explaining that *“It does not matter if you have all the money in the world. If you do not have a paper saying that you have a stable income, a permanent contract with a well-regarded company,* etc.*, they [the owners] will not rent you [the house or apartment] […].”*

As for the young people holding refugee status, by study design, they all shared a precarious housing situation. They had been recognised as beneficiaries of international protection (BIP) but were still housed in temporary reception centres, officially designated to accommodate applicants for international protection (AIP), due to the difficult housing market in Luxembourg. Common issues relating to the general overcrowding of the facilities were described by the great majority of the interviewees, such as the lack of privacy, issues of noise and cleanliness. These problems made life difficult not only within the reception centres but also outside of it, for example by interfering with their studies, as reported by Zula (female, aged 20, 2.5 years in Luxembourg): *“It’s difficult. […] For example, you live with three children in one [room], and you cannot study you do not have a quite [space], so you go to the [common room in the centre] and there are so many people that you cannot enter […] and they talk on the phone and they are loud.”* Yet, moving out of reception centres is very challenging due to lack of affordable housing. As Carim (male, aged 26, 6 years in Luxembourg), one of the only people interviewed with a stable job, explains *“[…] with my salary I cannot [move out]. […] they always refuse because I have a salary of 2000 euros. I found a flat that costs 850 euros a month and they said no because they want a salary of at least 2,500 or 2,700 euros.”*

### Labour market integration and the high cost of living

Even though Luxembourg has the highest gross domestic product *per capita* in the world, the economy is nowadays especially oriented towards the tertiary sector (e.g., financial industry) making it more difficult to find jobs in the industrial sector. Contrary to some migrants’ expectations, finding work in Luxembourg may not be an easy task. Joana (female, aged 29, 4 years in Luxembourg), from a Portuguese-speaking country, recalls that *“Everything was different from what I expected… first, I thought that I would immediately find a job upon arrival… it was the opposite, I had to wait one year.”* The high cost of living in Luxembourg is also a factor that takes some young migrants by surprise and has a huge impact on their lives. Carlos (male, aged 25, 5 years in Luxembourg), another migrant from a Portuguese-speaking country, says that life is more about surviving, reduced to the process of earning just enough money to pay the monthly expenses: *“It’s more about surviving… that’s what I’m doing […] the income, taking into account the cost of living, is not enough and to manage that is not easy […] the income is not bad but after paying for housing and monthly expenses, it does not pay off… things are very expensive here… and sometimes you have to think if it is worth it to stay here….”*

Overall, many of the participants considered finding a job one of the main challenges they (will) face in Luxembourg and a key aspect of their integration trajectories. Yet, the majority of the young people with refugee status interviewed were in educational programmes and only 3 were actively working at the time of the interviews, while few others had some sporadic work experience. Two of the people interviewed mentioned working without a regular contract. For one of them, his sporadic and non-regulated work in the construction sector presented a good opportunity to earn money, considering the limitations placed by law on student jobs^1^: *“Because black job [illegal work] is better. First [in formal jobs] they do not take me, because I do not have any experience. Second, I go to school, […] students have like a limited time to go to work. So black job is much better, there’s no taxes, so full for your money”* (Aref, male,_aged 19,_3years in Luxembourg). The money he earns with this type of jobs, however, is directed to support his extended family back in Afghanistan: *“Every penny, every euro because, yeah the thing is, my situation is much better than them. And they are struggling with the dinner and lunch and what they find to eat, even single bread. So this is really tough for them. I think every Afghani here is in situation like that, they have to send money to their families.”* Independently of whether they were employed or they were studying, the financial situation of the refugees interviewed was overall quite precarious.

### Social barriers: discrimination and racism

Perceived discrimination was reported in everyday life, within the educational system, public services, based on language, and as a manifestation of racism in all three groups but to different extents. Farid, one migrant with public visibility underlined that *“even in some administration like when you have different type of skin colour or it is obvious that you are non-European, you can feel it. Once again you cannot generalise it”* (Farid, male, aged 20, 8 years in Luxembourg). Reflecting further, he states that society in Luxembourg seems to be less open towards non-EU migrants: *“For Luxembourgish it is a weird nationalism - more like a European-nationalism. Because you have minority groups from other European countries like Portuguese, or other Europeans: French people, [people] from Belgium. I do not want to generalise but they repeat the same national phrases, but it is not towards other Europeans, but more towards third country nationals or those [who] have migrant background.“*.

Racism was also reported as a salient barrier to integration by some migrants from Portuguese-speaking countries. Helena (female, aged 25, 7 years in Luxembourg) and her father were reportedly told by a clerk and their municipal office *“you Cape Verdeans come here and you think you are in charge of everything.”* In another occasion, Helena says that when she once went with her husband to a doctor, was told *“you shameless people without papers [referring to her husband who was still waiting for the residence permit], you come here and even search for a doctor….”* The same interviewee additionally reported that such incidents are also common in public transportation means.

Contrariwise, discrimination and prejudice did not emerge as prominent issues among the refugee group as a whole, especially compared to what they experienced in other countries of residence. Yet, some participants did report feeling negatively judged, discriminated or stigmatised by the local population as refugees and described the overreaching and cumulative effect this may have on integration trajectories. As Aref (male, aged 19, 3 years in Luxembourg) explained: “*So that just delays getting me mixed in this culture and I was like… I was sometimes afraid that maybe the next person is going to treat me like that. So I was avoiding to talk and stuff like that.”*

Another commonly referred discrimination factor is based on *language* and associated with not speaking Luxembourgish. Rosa (female, aged 28, 4 years in Luxembourg), a migrant from a Portuguese-speaking country states that *“The biggest discrimination was based on the language… working for X company, I would always address customers in French and because I live in the North there are many Luxembourgers that only speak Luxembourgish or more Luxembourgish than any of the other languages and when they addressed me in Luxembourgish, I had to reply by saying ‘I’m sorry but may we speak French?’… often times their reactions were not the best [were not friendly]… and being stuck in a customer-employee relationship I could not react.”*

### Multilingualism

Language skills are perceived as central to the process of integration in Luxembourg, either for socialisation purposes, for work integration or for escaping discrimination. The country has three official languages: Luxembourgish, French and German. Each of these languages may play a significant role in the integration process depending on the region in which the migrant lives, the jobs he/she aspires to, the socialisation demands, access to public services. Participants of all 3 groups mentioned the multilingual situation as a barrier to integration. Joana (female, aged 29, 4 years in Luxembourg), a migrant from a non-EU Portuguese-speaking country, mentions that other people constantly tell her that *“you need to learn Luxembourgish to find a better job.”* She adds that *“I think that one of the things that prevents people from finding a job is the language [not knowing the languages spoken in the country].”*

Language was mentioned by 6 out of 7 young migrants with public visibility as an obstacle, at least at the beginning of the integration process. The multilingual context of Luxembourg with three official languages (French, German, Luxembourgish) makes linguistic integration specifically challenging. One participant even stated that it would be preferable to migrate to a country where migrants would need to learn only one language: “*I would recommend you going somewhere where there is only one language that even if you need to learn a new language, you have only one language to learn and you can also practice it.”* (Forough, female, aged 30, 10 years in Luxembourg).

Social exclusion may be one of the consequences of not dominating the multilingual Luxembourgish context and can be a barrier especially in the labour market which, depending on the sector, is either more oriented towards English (in research) or French (in private sector), or multilingual (public sector), but the multilingual context does play a role everywhere in everyday life: *“Language became an obstacle even though I have chosen to come to Luxembourg because [of the fact that people speak] English, but then I noticed that a lot of people do not speak English, or that they refuse to speak English, which is a different thing. And it was kind of a little bit disrespectful. Even in professional meetings, there are people who refuse to speak English. And they kind of use the multilingualism as an entry barrier rather than an integration thing “*(Azadeh, female, aged 30, 5 years in Luxembourg).

## Ingredients and triggers of resilient and empowering integration trajectories

When analysing the resources and strategies the young migrants perceive as effective in accordance with their resilient and empowering experiences, two main categories emerged: (1) individual and (2) relational/socioeconomic resources (see Table 3 for a summary of reported resources young migrants use, according to the groups under study).

**Table 3:** Resources mentioned by young migrants according to the groups under study.

Types of resources	Resources mentioned according to the groups under study
Individual resources	***Migrants from Portuguese-speaking countries and young migrants with public visibility*** E.g. self-efficacy beliefs, setting goals and determination.
Relational/ socioeconomic resources	***Migrants from Portuguese-speaking countries*** Family support, formal and informal community support. ***Migrants from Portuguese-speaking countries, refugees and young migrants with public visibility*** Support from a significant other. ***Young migrants with public visibility*** Support from teachers and professors, the supportive role of friends, the favourable socioeconomic background.

### Individual resources

Regarding individual resources, young migrants pointed out a host of agentic assets such as self-efficacy beliefs, having goals and the determination to accomplish them, courage, hope, entrepreneurship, social skills for networking and the acquisition of social capital, the developmental role of previous challenges, staying positive. For reasons of space, self-efficacy and setting goals and determination are given as examples of agentic capacities.

*Self-efficacy beliefs*. Self-efficacy beliefs were perceived as a powerful motivator to face and overcome integration challenges and achieve one’s goals. Tomé (male, aged 20, 10 years in Luxembourg), a young migrant from a Portuguese-speaking country, after having good results in an accountancy training course his father convinced him to attend (even though he himself was reluctant due to a perceived lack of language skills given the fact that he was still learning French), gained the conviction that he could actually move on to University: *“I was not expecting it but I did well in the accountancy training course… so I said to myself ‘I have the capacity to go to the University to do [study] this’ […], so I applied to several Universities […] and got accepted at the University of Luxembourg.”* One migrant with public visibility argued when reflection on some discriminatory experiences during pursuing her tertiary education in Luxembourg: “I am generally the way that if I hear people telling me ‘that you cannot do this, or this will not be possible’, these sentences just give me strength to do more and to become even stronger “(Forough, female, aged 30, 10 years in Luxembourg).

*Setting goals and determination*. Determination to persist with the integration efforts and reach one’s goals seems to be a key factor that motivates some young migrants to search for ways to overcome integration obstacles. Joana (female, aged 29, 4 years in Luxembourg), a migrant from a Portuguese-speaking country, asserts that *“I have a goal which is to find a job in my area of expertise… and that is one of the strengths I have.”* Francisco (male, aged 23, 3 years in Luxembourg), still a young migrant from a Portuguese-speaking country, says that *“We are young and we have to fight… I came from Brazil to Portugal [before coming to Luxembourg] but I did not leave my country to remain in the same situation… to leave implies a change in mind set… we leave to achieve our goals and to sacrifice ourselves… to make that exit worthwhile… it does not matter if it takes 2 or 5 years… we have to see the evolution, we cannot stand still in the same place.”*

Some refugees also mention determination as a way to cope with difficulties. Three participants explained it is important and useful to remain ambitious and keep having goals for the future when confronted with an obstacle or a problem, rather than settle. Hakim used these words of advice for newly arrived migrants in Luxembourg: *“they have to adapt themselves in the new society and I just ask them to not be disappointed, and to not be satisfied with something small and just take a big vision [for their future] go for it!”* (Hakim, male, aged_24, 2.5 years in Luxembourg).

On their turn, several migrants (3) with public visibility equally stressed the importance of their own motivation and determination: *“I guess it depends also on your personal motivation”* (Farid, male, aged 20, 8 years in Luxembourg). One other migrant with public visibility mentioned the possibilities in Luxembourg, but that nevertheless there might be difficult times and then it depends on your own motivation: *“Luxembourg is a country which offers many opportunities to people, and if you are really motivated and engaged, you can get everything that you want. It will not be easy, but if you want it and work hard enough you can get it”* (François, male, aged 31, 8 years in Luxembourg). Another migrant from the third group stressed determination to be independent as a goal as she argued that she could have had a better life financially speaking due to the socio-economic background of her family in her country of origin: *“My family, they are very rich, but I did not even want to ask my dad for money, I wanted to be independent. I wanted to do everything on my own, to stand on my own feet and to have a different mindset”* (Forough, female, aged 30, 10 years in Luxembourg).

### Relational/socioeconomic resources

Relational and socioeconomic resources mentioned by participants pertain both to the family and the community levels and have an iterative and interactive effect with the above-described individual resources.

#### Support from the family

The family often functions as a secure base from which the young migrant derives multiple resources such as mutual support (e.g., housing, financial support), trust, an environment for emotional expression, and the capacity to access external resources (profiting from the family’s social capital). Persistence, determination, will power to overcome integration challenges were sometimes described as coming from feelings of attachment to family members from which the young migrant receives emotional support and/ or motivation to accomplish his/ her goals and overcome integration challenges, but it also depends if the young migrants arrived in Luxembourg together with their family or alone. Helena (female, aged 25, 7 years in the country), a migrant from a Portuguese-speaking country, talking about the emotional and motivational parental support she receives in facing integration difficulties, says that *“If I did not have my mother in my life I do not know where I would be… she is a mother that talks with us and I always talk to her about everything… and she always gives me advice… ‘look my child do not be sad, here you’ll find work, your boyfriend will come here sooner’… […]. My mother is very open with us, she speaks with us about everything, I’ve always felt that strength she gives us… I’ve always had the support of my father also… but more from my mother….”*

The family can also be a source of instrumental support (e.g., provision of money, housing, mediation with social context). Instrumental support in housing has a huge relevance given the high housing market prices in Luxembourg. Rosa (female, aged 28, 4 years in Luxembourg), a migrant from a Portuguese-speaking country, living with her parents, attests the relevance of living with her parents in terms of economic savings and for obtaining a residence permit: *“[my family] helps me in terms of housing and having an address… because that is also a big problem for people who want to migrate… I came to my parents’ house and even so I had to prove that I was their daughter to stay here… in that sense I did not have much difficulty but I know people that did not make it, that did not find a way to get an address… and housing is very expensive… now it’s almost impossible to rent or buy… [prices] are exaggerated.”*

Finally, the socio-economic background of the participants, closely associated with their families’ social and economic capital, appears to be also decisive. While non-EU Portuguese-speaking migrants and refugees were facing conditions of vulnerability for which existing personal, familial and community resources did not seem sufficient to counteract them, migrants with public visibility seemed to benefit from the socioeconomic and cultural capital of their families. In fact, five out of seven migrants with public visibility mentioned that they came from a high socio-economic background with upper-middle class or wealthy parents in their country of origin. Several participants reported the good job positions of their parents and emphasised that they had a “rich life” in their country of origin and experienced a more privileged childhood (e.g., parents working in diplomacy) where they could obtain a good school education that helped them in their integration process in Luxembourg (e.g., already knowing French, German or English languages). This wealth may positively influence their childhood and youth development and provide them with levels of financial, social and cultural capital that facilitates their integration processes.

### Support from the community

#### Formal support

Support from the community can be either formal or informal. In terms of formal support social and employment services are relevant actors in supporting the integration efforts of young migrants. In Luxembourg citizens are entitled to a social inclusion income called REVIS (Revenu d’inclusion sociale, the Luxembourgish Social Inclusion Income) to provide a basic livelihood. However, specific conditions for third-country nationals exist (Art 2 and 3, 2018), who either need long-term residence status or need to have been legally resident for at least 5 years in order to receive it. People who have been granted international protection constitute an exception as they can benefit from REVIS immediately, while the Portuguese-speaking migrants need to have been living in the country legally for 5 years. Yet, REVIS is given only to people who are older than 25, which excludes part of our sample. In certain situations, formal support may assume a complex nature in order to conveniently address complex vulnerability conditions: Regina (female, aged 30, 11 years in Luxembourg), victim of domestic violence, along with her three children was given a place in a shelter home (a foyer). About the foyer where she is now living, she says: *“We are fine here, there are good conditions, we have everything we need… people here are great, very attentive, always asking if I need something… we are very well here, the kids are at school, we have a doctor, we have everything… they give me money to buy food […]. The only money that I have is what they give me.”* Talking about future help, she expects to receive, she says *that “As I still do not have a residence permit, I’m still not entitled to a family home [an apartment for the her and her children] and the subsidy I receive is not the Revis … it’s another type of help… as I do not have the residence permit.”* At the moment, she is waiting for the residence permit in order to be entitled to a family home and the Social Inclusion Income.

The Luxembourgish employment agency (ADEM, Agence pour le développement de l’emploi) was the most cited institution by non-EU Portuguese-speaking interviewees. These migrants sought support from ADEM for finding a job, receiving unemployment subsidies and for profiting from the language courses it promotes and finances. Manoela (female, aged 28, 5 years in Luxembourg), a migrant from a non-EU Portuguese-speaking country, reports that shortly after arriving, she felt the need to learn the languages spoken in the country, starting with French. In order to find French courses she could afford, she went to ADEM to take advantage of the low cost courses it provides: *“I went to ADEM because I did not have the money for the course [the French course taught in language centres] which is not that cheap… so I went there to find a way to pay less for the course.”* Carlos (male, aged 25, 5 years in Luxembourg), a migrant from a Portuguese-speaking country, about to lose his current job, aims to profit from the unemployment subsidy from ADEM he is entitled to: *“He [his boss] said that I might stay for two months with an unemployment subsidy… and if he [his boss] recovers again [the boss’ business] I might come back [to the same job].”*

#### Informal support

Informal support, on its turn, can assume many forms and come from local volunteers, neighbours, friends, religious communities, political parties, recreation centres, and other daily interpersonal relationships and was mentioned by several migrants in all three groups. A migrant with public visibility explained: *“I think that many people that I’ve met here played small roles. You could compare it to a big puzzle. Each piece represents someone that helped me or offered support. While living in the refugee camp, I met someone that was within politics. He helped me move out and stay with foster families for almost 2 years. A lot of people helped me with financial problems. Someone else helped me with finding a private teacher that tutored me in order to get my high school diploma. Another person helped me understand the Luxembourgish administrative procedures. I cannot pinpoint just one person that did everything”* (Francois, male, aged 31, 8 years in Luxembourg).

*Support of a significant other*. Particularly, the support of a significant other can have a powerful impact in overcoming the integration challenges both instrumentally (support in specific tasks) and emotionally (having someone that cares). Helena (female, aged 25, 7 years in the country), a migrant from a Portuguese-speaking country, when talking about her personal strengths and their source, mentions Manuel who works in an Association that supports youths’ work integration saying that *“I always say that he is my social assistant […], he gives me a lot of strength… I think that without him I could not do many things. Whenever I have a problem he helps me […]. I know him for 4 or 5 years […]. He always says ‘do not give up, you’ll manage to do that… so I always count on him because he helps me every time I need.”*

Upon arriving alone in Luxembourg at 17, Farah was paired with a family of long-term Luxembourgish residents, who volunteered to mentor her: “They are something like… they support me, you know? In everything. Not everything like for example money or not, no. Much more than money! […] We meet together for one time per week, but every week I have one class with her [*mother of the family is a French teacher] and I will say OK this week I did this one, this one, and she will advise me. […] She’s like my mother. And also the father of the family. I do not have a father now but I do not think [about that] because he’s such my father” (Farah, female, aged 19, 2 years in Luxembourg).

A migrant with public visibility also underlines the important role and support of one specific person who became *“like a father” [a person with a leading role in one of the big non-profit organisations in Luxembourg]. [That person] helped me a lot because this person was always beside me and behind me. […] He really directed me in Luxembourg. So he was really like a father for me here”* (Ahmed, male, aged 26, 9 years in Luxembourg).

*Support from teachers and professors*. As mentioned in the previous section, several participants explained how they felt a lack of support and even distrust or prejudice by teachers or the educational personnel in general. Yet, one migrant with public visibility explained: *“It depends really on your teacher if they just believe in you or they just see you as the foreigner who will have difficulties. So either the teacher can become a supporter or really the opposite”* (Ahmed, male, aged 26, 9 years in Luxembourg).

The examples below show that support from teachers also exists and can work as an important resilience factor supporting integration trajectories. Informal support from teachers and professors was highlighted by several migrants with public visibility as contributing to the resilience and empowerment of young migrants: *“There were teachers that voluntarily stayed there for 1–2 h, they did not get paid for that, to bring me at the same level as the others in the languages. […] I also got the support from my teachers, like I always had the chance to have best teachers who afterwards became my friends. I am still in touch with them. I think they saw something in me, so they helped me even when the school was finished or during weekends. I am still very grateful and that helped me to be where I am today”* (Farid, male, aged 20, 8 years in Luxembourg). At the university level, two migrants with public visibility mentioned the role of certain professors supporting them as the following example illustrates: *“One was my supervisor. He was really accommodating and, extremely responsive to what I needed”* (Prisha, female, aged 29, 4 years in Luxembourg).

*The supportive role of friends*. Several migrants with public visibility emphasised the important supportive role of friends: *“I think creating a good circle of friends who are interested in the same thing as you are”* (Azadeh, female, aged 30, 4 years in Luxembourg). “*[It is] very important to find a good group of friends. So, obviously, I did not know many people and it takes time to go from just knowing people to become good friends with them. And fortunately, I met a lot of people, and I became good friends with them overtime. And the support that you have with this friendship, it makes your personal life a lot easier and when your personal life is easier, your professional life becomes easier to handle too. And if there are some problems there so, for me, it was very important to have a good group of friends and I found them”* (Prisha, female, aged 29, 4 years in Luxembourg).

## Discussion

Taking into account the double transition of young migrants—simultaneously transitioning to adulthood and to a new socio-cultural milieu—the goals of the study were twofold: (1) to capture the processual cumulative effect of barriers to young migrants’ integration in the construction of conditions of vulnerability, in the period of coming of age; (2) to examine the ingredients and triggers of resilient and empowering integration and transition to adulthood trajectories. In doing so, integration trajectories of young migrants from Portuguese-speaking countries, young refugees and migrants with public visibility, all coming from outside the European Union, were analysed. Both vulnerability, resilience and empowerment of these young migrants seem to be a result of the processual interplay of structural conditions and agentic capacities over time, within the ongoing trajectories of liquid integration, at a time when they are making their transition to adulthood (Skrobanek and Jobst, 2019; Masten, 2021; Brodsky et al., 2022; Gilodi et al., 2022; Verbena et al., 2022; Gilodi et al., 2023). A double transition is therefore taking place (a migratory transition and the transition to adulthood), which adds complexity to the challenges these youths face and demand adequate resources to address them.

As is evident from the study of the transition to adulthood of young migrants in Luxembourg, such double transition may constitute a challenging journey, marked by legal barriers, housing problems, integration challenges into the labour market, experiences of discrimination and racism, and difficulties adapting to a multilingual landscape. These challenges intersect with existing economic, educational, social, and mental health dimensions, already common to many young non-migrants, amplifying the complexity of the transition process and often paving the way to paths of vulnerability.

### Paths to vulnerability

Barriers to integration, associated with insufficient resources to tackle them, usually lead to the formation of conditions of vulnerability—a lack of power to conveniently address and overcome such conditions. Often times, a cumulative effect occurs, when intersecting barriers are followed or lead to others in a sequential or even a systemic pattern that increases integration challenges and aggravates experienced conditions of vulnerability. Usually, structural barriers to integration become the trigger of a sequential process of cumulative or interdependent factors that may ultimately lead to subjective feelings of vulnerability materialised in a sense of helplessness (as theorised by Seligman, 1991) and hopelessness (as explored by Beck et al., 1974). Extreme uncertainty, feeling stuck, isolation, fear, distress, liminality experiences, social withdrawal, anxiety and depressive symptoms may be the ultimate consequences of negative experiences in several life domains (e.g., Carruth et al., 2021; Hartonen et al., 2022).

Observed structural barriers to the integration process emerged from the analysis are manifold and multidimensional: legal or bureaucratic hurdles, difficulties in finding adequate housing arrangements, challenging school and work integration, the high cost of living in the country, perceived discrimination (e.g., based on language, country of origin, race), absence of family support, lack of adequate language skills in a country that poses the challenges of multilingualism, social isolation. For refugees, there are the added defies of long stays in refugee reception centres (Hartonen et al., 2022, 2023).

As reported previously, conditions of vulnerability seem to build up in the form of clusters of individual, situational and structural factors, according to an intersectional understanding of vulnerability (Gilodi et al., 2022; Siller and Aydin, 2022). The first of those reported factors consist of legal barriers, mentioned by the two vulnerable groups under study: migrants from non-EU Portuguese speaking countries and refugees. As the interviews from the first group (migrants from non-EU Portuguese-speaking countries) showed, one participant arrived in Luxembourg without papers, and therefore experienced strong legal barriers influencing all other aspects of his life leading to a fearful isolated life, not being able to legally entering the labour market, and finally to the development of feelings of humiliation, hopelessness, and the emergence of anxiety and depressive symptoms. But also other participants with regular residence permits mentioned legal barriers (e.g., recognition of diplomas) influencing their integration trajectories and experiences. In the case of refugees, while waiting for the unfolding of the asylum process, they were left in a sort of a limbo (as found in previous literature, e.g., Hartonen et al., 2022, 2023), with their rights severely limited (e.g., the right to cross borders, limited access to the labour market). Such experience, when combined with the protracted stays in reception centres, may have lasting effects even after receiving refugee status (as all the participants interviewed in group 2), affecting their capacity to envision their future and thus stalling or protracting their transition to adulthood.

Finding of affordable housing is another major situational hurdle young migrants face in Luxembourg, affecting all three groups under study but especially migrants from non-EU Portuguese speaking countries and refugees. On the one hand, the high rental or property prices become prohibitive for young migrants with insufficient financial resources. On the other hand, the usually demanded guaranty of a permanent job contract and a considerable income level (as a payment guaranty) can seldom be provided by young migrants who, at the beginning of their work careers and their integration trajectories, are especially prone to more precarious job conditions (e.g., fixed-term contracts) and lower wages. In such a context, support from the family in terms of providing housing and financial assistance becomes extremely relevant. In the absence of such support, young migrants from Portuguese-speaking countries reported struggling to make ends meet, having the experience of daily wrestling to merely survive, often working in irregular conditions, feeling stuck in a life situation experienced as profoundly unsatisfying and with no promise of a better future in sight.

Young refugees, on their turn, housed in temporary reception centres, struggle with difficult living conditions in the centres which often involve, for example, having to deal with lack of privacy, appropriate space for intimacy and activities such as studying. The limiting effects of such housing conditions on the normal unfolding of life, in a life stage where they are making their transition to adulthood, can have a relevant impact in this phase of their lives and for their future: the needed exploration of possible paths of transitioning to the world of work, finding financial independence, establishing romantic relationships, and slowly developing an adult identity is severely curtailed, with their adult development being put on hold. Moreover, their wider social integration in the Luxembourgish society may be delayed and made more difficult due to a lack of a regular contacts and interchanges with the local population in all spheres of life. Such waiting experience in reception centres seems to lead to experiences of indeterminate liminality, with the consequent need to cope with a sometimes a long transition phase before ulterior reaggregation into society (Turner, 1969; Alkhaled and Sasaki, 2022).

For many of these young migrants, situational challenges such as the difficult entering into the labour market, coupled with the high cost of living in the country and the limited recognition of diplomas earned in the origin country, poses yet another major challenge to the unfolding of personally satisfying and meaningful transition to adulthood trajectories. This situation also especially affects migrants from non-EU Portuguese speaking countries and refugees. Having to settle for precarious, short-term jobs with insufficient earnings is the lot of many. Others (as the case of some refugees), feel the obligation of sending their sparse earnings to family members that stayed in the home country in order to financially support them and consequently managing to survive with several forms of financial aid provided by the State. Usually, in order to improve their financial situation by gaining access to better job positions, a long, complex and labyrinthine path ensues: conjugating precarious jobs with further studies (e.g., studying the official languages of the country, carrying out a vocational training), slowly developing the necessary social capital to widen job prospects. Generally, these young migrants have to endure long years with reduced financial well-being while maintaining a glim sense of hope of better future prospects. Overcoming such hurdles and finally arriving at a position where they are publicly recognised in a certain professional field, usually consists of a long and complex journey paved with strenuous work coupled with difficult access to resources, as reported by young migrants with public visibility.

Given the complex challenges young migrants face in their process of coming of age, family support (as previously stated), in several forms (e.g., housing, financial, emotional support), was found in previous research (e.g., Oliveira et al., 2014; Cano-López et al., 2021), as being of paramount importance to provide young people with a secure base (both emotionally and economically) and the necessary instrumental aid that is indispensable to help them overcome the huge challenges they face. This was partially confirmed by our data, where some participants from non-EU Portuguese speaking countries reported the important role their families played in supporting them. When such support is absent or insufficient, some young migrants are left on their own (or with access to mere instrumental support from the State) to face the transition to adulthood challenges in a context (that of migration) that usually exponentially aggravates such challenges (Willems et al., 2015). Indeed, family support was not readily available for those participants who arrived to Luxembourg alone, who, for the most part did not see this as problematic. In other cases, family members were present and ready to support our young participants, but their own lack of power and resources meant that their support, especially in financial and material terms, was also limited. When lack of family support is clustered with other conditions of vulnerability, as observed, a complex risky situation may emerge to the point of negatively affecting the young migrant at a core psychological level. As an example, when family conflicts pushed the young migrant out of the family home (and ceased being the source of any other kind of support), there was the need for the young migrant to enter the difficult path of finding his/ her own housing arrangements. If he/ she managed to find a place to live, given the high rental prices in the country, a huge fraction of his/ her usually sparse earnings will be spent on the rent. The consequence may be the struggling to make ends meet, and again having the reported experience of being merely surviving. Here, again, we can observe the cumulative and intersectional effects (Gilodi et al., 2022) of detrimental situational factors at work that may have relevant negative consequences at a core psychological level, such as the observed feelings of helplessness and hopelessness, from which the development of anxiety or/ and depressive symptoms emerged.

A further hurdle that may impose additional complexity to the young migrants’ integration processes and have a significant impact on the process of transition to adulthood stems from the confrontation with socially discriminatory practises. This may be observed in several contexts, from school to work settings, in public services and wider social interactions and was especially reported by young migrants from Portuguese-speaking countries and some migrants with public visibility. Within the school system, especially when transitioning from primary to secondary school, migrant children are often driven away from classical studies (opening the way to subsequent University studies) and pushed into technical or vocational studies. a. Such a discriminatory practise may have a profound impact on school-to-work transition trajectories and even on adult life trajectories. The effects of such a phenomenon may lead to the production of a process of social segmentation (e.g., Martin, 1994; Meyer and Vasey, 2020). On the one hand, young migrants affected by such discrimination are driven to less prestigious and less well-paid jobs, a factor that may pose additional difficulties to the school-to-work transition and to the process of transition to adulthood in general, as it would negatively impact the conditions of constructing a financially independent, adult way of life. On the other hand, such school and subsequently professional segmentation inevitably produces a further process of social segmentation and differentiation based on socioeconomic and cultural statuses. Here we are observing the intersectional production of conditions of vulnerability (Gilodi et al., 2022) that can have an impact on future generations, in the form of a social reproduction of inequalities (Spire, 2015).

Racism was another form of discrimination especially reported by young black migrants from non-EU Portuguese-speaking countries. Racism was experienced by these migrants in diverse contexts: in public services, medical appointments, public transportation. The deleterious effects of such pervasive practises also materialise in a non-egalitarian and non-inclusive society that (re)produces processes of social segmentation (Spire, 2015; Finney et al., 2023). The ultimate effect on victims of racism is that they end up living in a society that fails to fully welcome and integrate them as full right citizens benefitting from equal social treatment as their peers, by negatively singling them out as a possibly undesirable social group, thus paving the way to situations of vulnerability. Comparatively, young refugees overall reported fewer instances of prejudice, discrimination or racism, but some experiences of subtle (or in few cases blatant) discrimination and stigmatisation based on negative stereotypes and prejudices against their cultures of origin or against refugees did emerge. Such experiences may lead to a process of stereotype embodiment (Levy, 2009) and induce on the part of the young migrant a behaviour of avoidance of social contact due to fear of being a victim of discrimination, a factor that inevitably induces feelings of social isolation and impairs these young migrants’ process of liquid integration (Skrobanek and Jobst, 2019). In the case of refugees, our data show how feelings of social isolation were heightened by living in reception centres with other migrants which limited their interaction with long term residents of Luxembourg.

Discrimination was observed also with respect to languages. Not speaking the official languages of the country, especially Luxembourgish, may constitute a base for discrimination. A situation especially reported by migrants from non-EU Portuguese speaking countries. The Luxembourgish language seems to be a door opener to smoother social interactions with the Luxembourgish population in several contexts, from work to the wider social milieu. The only use of one of the other official languages (especially French), may impair communication and stimulate discriminatory practises.

Still, with respect to languages, Luxembourgish multilingualism also poses additional integration barriers in several domains such as school, work and social integration, especially for migrants from non-EU Portuguese speaking countries. Our analysis suggests that failing to proficiently acquire language competences in at least two of the three official languages of the country can lead to cumulative effects such as difficulties in following school curricula, being excluded from certain job positions or experiences of social exclusion within relational contexts.

### Paths to resilience and empowerment

Resources and strategies needed for resilience and empowerment tend to substantially overlap, according to the TIMER model, although the focus of change in resilience is internal, whereas in the case of empowerment, such a focus is external (Brodsky and Cattaneo, 2013; Brodsky et al., 2022). Accordingly, processes of resilience and empowerment will be discussed jointly and their distinction made evident when dealing with the focus of change.

As observed in the building of vulnerability trajectories, here again the construction of resilient and empowering paths tends to present a multidimensional and systemic configuration, involving the intersecting of individual, familial and community resources (Masten, 2021; Brodsky et al., 2022). When communicating individual resources and strategies, all groups of migrants under study referred a long list of agentic capacities needed to face and overcome integration risks and challenges: self-efficacy beliefs, self-esteem, self-reliance, setting goals and determination to accomplish them, courage, hope, being positive, entrepreneurship, the ability to establish social contacts, incorporating the resilience assets developed in the course of previous life challenges (especially for young refugees), the capacity to fight stereotypes, adjusting expectations, and the acquisition of knowledge and education. Internal resilience denotes the presence of identity capital and a motivation and capacity to mobilise internal resources to face contextually situated needs, risks and challenges (e.g., Côté, 2006, 2014). The young migrant is thus internally mobilised to explore resilient ways to move forward in his/ her integration trajectory in order to overcome experienced challenges especially when external support is lacking. Indeed, some young migrants (especially refugees), in the felt absence of other immediate sources of support seem to have fallen into a self-reliance mode of coping with experienced problems and difficulties. The more there is a felt lack of structural support (social, relational or institutional), the more the mobilisation of internal resources (and the ultimate self-reliance mode of approaching difficulties) becomes the main resilience strategy—one can count but on oneself (e.g., Côté, 2006, 2014). Yet, whether individual coping strategies in the face of challenges will result in long-lasting resilient strategies, leading to the desired position in society and the fulfilment of personal ambitions or will become maladaptive and lead to harmful cognitive and behavioural patterns cannot be determined through a cross-sectional study.

Mobilising internal resources may be more efficient as a resilient and empowering strategy when conjugated and interacting with contextual assets and resources (Masten, 2021). The latter can come from the family or from the community at large. As reported especially from young migrants from non-EU Portuguese-speaking countries, family support assumes a most relevant role in assisting the young migrants’ coming of age in a complex migration context that poses numerous challenges. First and foremost, the family may serve as a secure base, providing emotional support, motivation and encouragement. In this sense, it serves as a social anchoring (Grzymala-Kazlowska, 2016) of the integration process. Indeed, several migrants report that without such support from their family, they would not have been able to cope with the integration challenges they faced. Yet, the family may also provide certain types of instrumental support that are of paramount importance in this life phase of young migrants. Having such instrumental family support may liberate the young migrants to focus their energies and resources (many of the individual resources mentioned above) to the pursuit of other goals that surpass the mere survival level. They can instead concentrate on actually flourishing in areas of their choice (e.g., improve their educational level; explore different work opportunities according to their vocational interests). In fact, the socioeconomic level of the family can have a decisive role in allowing transitioning from processes of resilience to processes of empowerment. As seen especially in the case of young migrants with public visibility, the relatively high socioeconomic statuses of their families seem to have provided them the means (in conjugation with agentic capacities) to have integration trajectories with an external social impact, making them socially visible in areas such as entrepreneurship and in the academic domain. On the one hand, the economic, social and cultural capital that usually go hand in hand in families of higher socioeconomic levels play a crucial role in liberating young migrants from concentrating merely on resilience strategies. On the other hand, such family capital may grant them the conditions to focus on the development of life projects that, over time, may ascribe them the possibility of producing a positive and visible social impact, providing them professional and social notoriety. Here again, a process of social reproduction can be observed with the intergenerational transmission of different forms of capital (e.g., economic, social, cultural) that can have a profound impact on the empowerment of young migrants (Bourdieu, 1977; Bourdieu and Passeron, 1977; Weiss, 2021). At the same time, it is also important to mention that the migrants with public visibility have already been living in Luxembourg for a longer period of time and therefore have overcome already some of the integration challenges experienced just after arriving in the country. Some arrived in Luxembourg as asylum seekers (3 participants out of 7) but they have been living in Luxembourg for a minimum of 6 years. This temporal component needs to be taken into account when analysing the unfolding of the liquid integration trajectories of young migrants.

Another relevant form of support comes from the host community. Such support can be either formal or informal. Formal support was especially mentioned by migrants from non-EU Portuguese speaking countries, whereas informal support was highlighted especially by migrants with public visibility. Institutionalised support systems (e.g., provided by State agencies, associative services) pursue country policies aimed at instrumentally helping migrants’ integration in a host of different areas such as employment, housing, providing subsidies, delivering language courses. In many instances, such support is fundamental as a way to provide minimal conditions that prevent some young migrants from falling into situations of utter helplessness, when all other sources of support are absent or prove insufficient. Concomitantly, formal support serves as an anchor that allows young migrants to have sufficient survival means that allow them to slowly start developing the conditions needed to engage in developmental coming of age trajectories that ultimately can lead them to the acquisition of autonomy and an independent adult way of life. In Luxembourg, one important type of formal support constitutes the attribution of a “social inclusion income” (REVIS).^2^ Yet, such subsidy is attributed according to certain criteria (Art 2 and 3, 2018). Entitled to REVIS are third-country nationals who are neither stateless persons nor beneficiaries of international protection who have been legally resident in Luxembourg for at least 5 years (continuously or not) within the past 20 years; or have long-term resident status. The 5-year residence condition does not apply if the third-country national is a family member of a Luxembourg citizen, an EU national, a national of the European Economic Area or Switzerland or a person who has been granted international protection. Accordingly, refugees can benefit from REVIS immediately while the non-EU migrants from Portuguese-speaking countries need to have been living in the country for 5 years. Yet, the REVIS is given only to people who are older than 25, which excludes part of the participants in the study, independently of the group to which they belong. As a consequence, young migrants aged between 18 and 25 years who need financial assistance may be more prone to financial vulnerability since they are not eligible to receive “social inclusion income.”

Apart from formal support, community informal support can also have a profound impact on the development of resilient and empowering life trajectories. Such support, especially highlighted by migrants with public visibility, may be more diverse and be expressed in a multitude of ways. Yet, it usually involves the establishment of a significant connection with individuals from the local population that promotes feelings of being welcomed or helps in the instrumental solution of integration defies. This kind of support can come from teachers, professors, neighbours, friends, acquaintances. It may assume the guise of emotional support, motivation, encouragement or instrumental help. Especially relevant seems to be the establishment of a relationship with someone that becomes a significant person in the young migrant’s life—someone with whom an attachment bond is formed. On the one hand, the accumulation of informal support may contribute to the establishment of a close social network that promotes the anchoring of the young migrant in the host society and from which he/ she may start developing a sense of belonging (Grzymala-Kazlowska, 2016). On the other hand, such anchoring may constitute a privileged mean to develop one’s social capital and thus enhancing the needed social resources for the implementation of resilient and empowering trajectories. With respect to empowering processes, migrants with public visibility mention the extreme relevance of close relationships with friends and the development of attachment bonds with significant others in their lives. Usually, they credit these fortunate encounters with the success of their endeavours that ultimately had the social impact that made them socially visible and notorious.

### From vulnerability to resilience and empowerment within the double transition of young migrants

Following previous literature (Gilodi et al., 2022, 2023), this paper showed that vulnerability paths are often a consequence of negative intersectional elements from various structural and situational dimensions (political, economic, cultural, social, personal) for which the young migrant does not possess sufficient power (agentic capacities, socioeconomic and cultural resources) to counteract its effects. Instead, the resilient and empowering paths are often a consequence of the existence of internal resources and external support mechanisms. Yet, this also must be seen from a life-course perspective, therefore we argue for the liquid integration trajectories emphasising also the dynamic, processual, and temporal nature of migrant integration trajectories. The interviewees of group 3 (migrants with public visibility) were partly already longer living in Luxembourg and therefore, with time, managed to overcome certain integration barriers.

As observed many intersecting integration obstacles (legal barriers, access to housing, integration into the labour market and the high cost of living, insufficient family support, social barriers such as discrimination, multilingualism) act to produce conditions of vulnerability and ultimately make the process of transition to adulthood more complex and riddled with risks for the psychosocial development and well-being of many young migrants coming to Luxembourg from outside the EU. In such conditions, the process of identity exploration that became typical of youths in their twenties (e.g., Arnett, 2000, 2015; Côté and Levine, 2014) may become severely constrained under conditions that limit the necessary exploration of constructive and developmental paths to adulthood. Oftentimes, young migrants lacking enough resources or support for the challenges they face in their coming-of-age trajectories end up in a situation where their lives are put on hold. For some economic migrants this results in an experience of being merely surviving, as opposed to an experience of growth towards the attainment of personally meaningful and desired life goals. For refugees, their experience of waiting in reception centres tends to produce experiences of indeterminate liminality, with the consequent need to cope with a sometimes long transition phase before ulterior reaggregation into society (Turner, 1969; Alkhaled and Sasaki, 2022). In such situations, what should be the normal unfolding of life and the normal psychosocial development of these young migrants is severely curtailed by the integration difficulties they face. Their levels of well-being are consequently lowered, sometimes to the point of developing severe anxiety and depressive symptoms, usually when experiences of helplessness and hopelessness set in (Beck et al., 1974; Seligman, 1991).

Many difficulties faced by young migrants in vulnerable situations are also presently widely experienced by young non-migrants in many countries, given the complexities and difficulties inherent to the process of transition to adulthood. In fact, it was this acknowledgment, more than two decades ago, that led Côté and Allahar (1996) to speak of a “generation on hold,” that is, a young generation being kept from normally moving on with their lives, on account of structural factors (especially associated with difficulties in entering the world of work and obtaining a decent earning) that make it extremely difficult to achieve the financial conditions needed to assume adult roles and responsibilities. Yet, as observed in the present study, young migrants may face even more difficult and complex conditions of transition to adulthood, given the intersection of a wider array of multi-layered challenges that may come not only from difficulties in making a positive or optimal school-to-work transition, but also from other sources such as legal barriers, difficulties in accessing decent housing, language adaptation, and difficulties in sociocultural integration stemming, among other factors, from discriminatory practises. It is such a complex confluence of difficulties and challenges, coupled with often insufficient or inadequate support systems, that usually leads to the formation of conditions of vulnerability that are specific of young migrants.

Such a systemic confluence of multi-layered conditions of vulnerability inevitably demands a systemic response leading to the production of the necessary conditions for the production of resilient and empowering transition to adulthood trajectories for young migrants, as already observed by Gilodi et al. (2023). The latter stem from the confluence and dynamic interaction of structural resources and agentic capacities (Masten, 2021). Instead of looking individually and in an isolated form to specific factors (inductive of resilience and empowerment), such factors and their effects may be better understood when dynamically clustered. Only then, their dynamic interplay is made salient and the processual nature of their functioning is made visible. Looked at from this angle, vulnerability and empowerment can be portrayed as the two poles of a continuum. The vulnerability pole exemplifies the negative power imbalance of challenges and available resources. In such a case, the young migrant is overwhelmed by difficulties for which he/ she does not possess the matching assets to counteract them. On its turn, the empowerment pole exemplifies the optimal manifestation of power resources in the face of existing challenges, to the point of allowing the migrant to produce change in the external social milieu (Brodsky and Cattaneo, 2013; Verbena et al., 2022). Resilience would stand as a middle term between vulnerability and empowerment, where young migrants manage to positively face experienced challenges but, nevertheless, have not still come to the point of having an external social impact on the milieu they are conducting their lives. Looked at from this perspective, when in situations of risk, vulnerability could be added to the existing TMER model as a way to fully approach resilience and empowering trajectories stemming from existing conditions of vulnerability. Only then could we have a comprehensive perspective on vulnerability, resilience and empowerment.

Hence, in order to tackle vulnerability conditions and induce processes of resilience and empowerment, a systemic approach must be adopted. While acknowledging the relevant role of agency in navigating increasingly individualised transition to adulthood trajectories (Côté and Levine, 2014), within a context of liquid migrant integration (Skrobanek and Jobst, 2019), it is indispensable to promote adequate formal (institutional) and informal (anchored in social relationships) support systems apt to induce the resilience and empowerment of young migrants. In the case of Luxembourg, the findings of this study suggest that an important part of the work needed to eliminate structural barriers to the integration of young migrants and contribute to fostering resilient and empowering transition to adulthood trajectories should come from the socio-political sphere. Policies and measures addressing problems that hinder favourable young migrants’ transitions to adulthood trajectories highlighted by the study could include: rendering social housing more easily accessible; fighting discriminatory practises in several spheres of public life (in schools, public services and society at large); providing alternative accommodation for refugees in reception centres; extending the social inclusion income to migrant youths under the age of 25; strengthening support interventions for positive school-to-work transitions; adopting measures aimed at reducing the integration barrier of multilingualism (e.g., promoting more flexibility in the use of the official languages of the country by not assuming proficiency in the three official languages as a prerequisite for optimal school, work or social integration). Such measures could have a positive impact on young migrants’ integration in the country. On the one hand, they could help foster more personally satisfying and fulfilling transition to adulthood trajectories with the associated benefits stemming from the full socioeconomic and cultural contribution of this younger generation of migrants. On the other hand, society as a whole could start avoiding the present and future social costs of the possible marginalisation of a generation of young migrants and contribute to the construction of a more equitable and inclusive society.

Conclusion: Liquid integration and the construction of vulnerability, resilience and empowerment within the double transition of young migrants.

According to the liquid integration perspective (Skrobanek and Jobst, 2019), the integration of young migrants in their process of coming of age is a fluid, dynamic and multifaceted phenomenon that entails the interplay of structural factors and individual experiences, over time. Time is of primordial relevance for the liquid integration perspective as it consubstantiates the frame upon which the cumulative effects of the integration dialectics between challenges and resources is played out.

When difficulties (e.g., legal barriers, housing insecurity, discrimination, racism, and multilingualism of the host society) weigh more than available resources (personal or social), the dialectical pendulum tends to push the migrant to a path of vulnerability: weaknesses accumulate and systemically articulate to corner the individual into a living position where an experience of insufficiency to face existing integration challenges becomes the trend. Two situations explored earlier may serve as examples: (1) difficulty in finding decent and affordable housing arrangements due to discriminatory practises in the housing market, lack of affordable housing options, and overcrowded living conditions can contribute to social exclusion and marginalisation, with possible ensuing homelessness or precarious housing situations further undermining migrants’ ability to establish autonomous, stable adult lives and participate fully in their new communities; (2) systemic discrimination in employment, education, and social services perpetuates inequalities and erodes migrants’ sense of belonging and social cohesion, for these experiences not only harm individuals’ well-being but also hinder their opportunities for socioeconomic advancement, exacerbating vulnerabilities and perpetuating cycles of marginalisation in the process of establishing an adult status.

Within the continuous unfolding of the liquid integration process, the cumulative effects of individual resources such as self-efficacy, goal setting, determination, as well as social resources including family support, community networks, and socioeconomic resources, can also promote resilient integration trajectories among young migrants, even among those previously trapped in vulnerability paths. The triggers of a resilience movement may be found both in individual and social resources. Individual resources may play a crucial role in shaping migrants’ resilient integration experiences and outcomes when transitioning to adulthood. Self-efficacy, or one’s belief in the ability to accomplish tasks and overcome challenges, serves as a fundamental determinant of success in adapting to new environments. As an example, migrants with higher levels of self-efficacy are more likely to persevere in the face of obstacles, actively seek out opportunities for growth, and effectively navigate unfamiliar contexts, thereby promoting resilient integration trajectories. In addition to individual resources, social resources may also play a vital role in supporting migrants’ integration journeys. Family support serves as a critical source of emotional, practical, and financial assistance, buffering against the stressors and challenges associated with migration in the period of coming of age. Strong family ties provide a sense of belonging and security, fostering resilience and empowering migrants to overcome obstacles and achieve their goals. Within the social triggers of resilience, formal and informal community support networks also play a significant role in promoting resilience. Access to community resources, such as language classes, job training programmes, and cultural orientation workshops, facilitates social integration and provides valuable support to migrants as they navigate unfamiliar systems and institutions. Informal networks, including friendships, religious communities, and ethnic associations, offer social support, companionship, and a sense of belonging, contributing to migrants’ well-being and integration success in this crucial phase of their lives.

Lastly, socioeconomic resources, including access to education, employment, and financial stability, seem to play a critical role in facilitating empowered integration paths. Economic empowerment enables migrants to achieve greater independence, secure stable housing, and participate fully in their new communities, further fostering resilience and empowerment, as they navigate their transition to adulthood trajectories.

The differences between the migrant groups under study (migrants from non-EU Portuguese speaking countries, refugees and migrants with public visibility) stands as evidence of differences in available psychosocial and economic resources to tackle experienced integration challenges. As is evident by now, in the liquid integration process of the migrants under study, individuals navigate two complex transitions (transition to adulthood and the integration in a new country) shaped by their personal and social resources, leading to diverse trajectories of adaptation and agency. Some young migrants, lacking sufficient resources to counter experienced barriers and challenges, enter vulnerability paths, while others, leveraging psychosocial resources, construct resilient integration trajectories. Still, another group, drawing on available socioeconomic resources, embark on empowered integration paths, enabling them to assume adult responsibilities and have a transformative impact on their communities. Firstly, migrants on vulnerability paths face significant challenges and barriers to integration due to a lack of personal and social resources. These individuals often confront systemic inequalities, discrimination, and limited access to essential services and support networks. Their vulnerability stems from socio-economic disadvantages and language barriers, hindering their ability to adapt and thrive in their new environments. Vulnerable migrants may experience social isolation, economic insecurity, and heightened stress, making it difficult for them to access opportunities for advancement and participate fully in their communities. In contrast, migrants on resilient integration trajectories demonstrate the capacity to overcome adversity and construct meaningful adult lives in their host countries. These individuals draw on psychosocial resources such as determination, and social support networks to navigate challenges and setbacks. Resilient migrants tend to exhibit a positive mindset, adaptability, and problem-solving skills, enabling them to bounce back from setbacks and maintain a sense of well-being amidst adversity. They may engage in self-care practises, seek out supportive relationships, and actively pursue opportunities for personal growth and development, contributing to their resilience and integration success. Lastly, migrants on empowered integration paths (especially exemplified by migrants with public visibility) leverage their socioeconomic resources to not only adapt to their new environments but also to effect positive change and transform their communities. These individuals have acquired sufficient financial stability, access to education and employment opportunities, and social capital, enabling them to exert influence and agency in their social and political contexts. Empowered migrants are able to advocate for their rights, challenge systemic injustices, and engage in collective action to address community needs and promote social justice and inclusion. They may initiate grassroots initiatives, participate in civic engagement activities, and serve as catalysts for positive change, fostering inclusive and resilient communities where all individuals can thrive. In summary, the lived experiences of these three groups of migrants underscore the complex dynamics of the integration process and the importance of personal and social resources in shaping individuals’ transition to adulthood trajectories. While vulnerability paths represent struggles and barriers to integration while transitioning to adulthood, resilient integration trajectories highlight individuals’ ability to overcome adversity and maintain a sense of well-being. Empowered integration paths, on the other hand, demonstrate migrants’ capacity to not only adapt but also to effect transformative change in their communities.

In conclusion, the liquid integration perspective underscores the interconnectedness of integration difficulties and their cumulative impact on young migrants’ vulnerabilities. Addressing these challenges requires comprehensive and inclusive policy interventions that prioritise social justice, equality, and human rights. Efforts to dismantle legal barriers, promote affordable housing, combat discrimination, and facilitate language acquisition are essential for fostering inclusive, resilient and empowered communities where migrants can thrive as accomplished adults. By recognising the intersectionality of integration difficulties and their implications for vulnerabilities, policymakers, researchers, and practitioners can work collaboratively to create more equitable and supportive environments for young migrants, thus favouring the promotion of resilience and empowerment in the process of transition to adulthood. Even though resilience and empowerment can both be seen as systemic processes, empowerment seems to demand a greater capacity to engage with collective processes that involve articulated actions with others, able to challenge existing power structures and norms. Empowerment is fostered through supportive relationships, access to resources and opportunities, and participation in decision-making processes. It requires individuals to critically reflect on their own identities, values, and goals, and to take collective action to address systemic injustices and inequalities. In this sense, empowerment is inherently linked to social change and transformation, whereas resilience may be more focused on individual adaptation and coping strategies. In summary, while resilience and empowerment are both important concepts in understanding how individuals navigate integration challenges and transitions, they represent distinct pathways towards adaptation and agency. Resilience is about bouncing back from adversity and maintaining stability in the face of challenges, whereas empowerment involves gaining control over one’s life, challenging systemic barriers, and effecting positive change. By recognising the differences between resilience and empowerment within liquid integration and transition to adulthood trajectories, we can better understand the complex dynamics of resilience-building and empowerment processes, and work towards creating more inclusive and equitable societies where all individuals can thrive.

As for the study’s limitations and suggestions for future research on this subject, a follow-up of the participants’ integration and coming of age trajectories would allow to better grasp the evolution of the double transition they are facing. As in any systemic process, time is a fundamental frame to adequately capture the manufacturing of such trajectories, as the time of stay in the country represents a significant factor at play in integration trajectories. Future longitudinal studies would also be the adequate means to further and more deeply capture the dynamic and systemic interplay of factors that over time contribute to the development of trajectories of vulnerability and resilience/ empowerment in the context of migration.

## Data availability statement

The raw data supporting the conclusions of this article will be made available by the authors, without undue reservation, to researchers upon reasonable request. However, due to the sensitive nature of the data collected from vulnerable populations, stringent measures have been implemented to protect the confidentiality and privacy of participants. Access to the data will be granted in compliance with applicable ethical and legal regulations, ensuring that the rights and well-being of the participants are safeguarded.

## Ethics statement

The studies involving humans were approved by the Ethics Review Panel, University of Luxembourg. The studies were conducted in accordance with the local legislation and institutional requirements. The participants provided their written informed consent to participate in this study. Written informed consent was obtained from the individual(s) for the publication of any potentially identifiable images or data included in this article.

## Author contributions

BN: project administration and funding acquisition. JO, JB, AG, and CR: data collection. JO: writing—original draft. All authors contributed to the article and approved the submitted version.

## Footnotes

^1^By law students enrolled in non-vocational training are not allowed to work more than 60 h a month: https://guichet.public.lu/en/entreprises/ressources-humaines/contrat-convention/jeunes-actifs/contrat-etudiant-cdd.html.

^2^Criteria for the attribution of REVIS can be consulted here: Applying for social inclusion income (REVIS)—Citizens—Guichet.lu—Administrative Guide—Luxembourg (public.lu).
